# Cardiopulmonary Exercise Testing in Lung Transplantation: A Review

**DOI:** 10.1155/2012/237852

**Published:** 2012-05-14

**Authors:** Katherine A. Dudley, Souheil El-Chemaly

**Affiliations:** ^1^Harvard Combined Pulmonary Fellowship Program, Harvard Medical School, Boston, MA 02115, USA; ^2^Division of Pulmonary and Critical Care Medicine, Brigham and Women's Hospital, Harvard Medical School, Boston, MA 02115, USA

## Abstract

There has been an increase in lung transplantation in the USA. Lung allocation is guided by the lung allocation score (LAS), which takes into account one measure of exercise capacity, the 6-minute walk test (6MWT). There is a paucity of data regarding the role and value of cardiopulmonary stress test (CPET) in the evaluation of lung transplant recipients while on the transplant waiting list and after lung transplantation. While clearly there is a need for further prospective investigation, the available literature strongly suggests a potential role for CPET in the setting of lung transplant.

## 1. Introduction

### 1.1. Lung Transplantation in the United States

 There are approximately 1,700 patients listed for and awaiting lung transplantation in the United States [1]. Patients with underlying idiopathic pulmonary fibrosis (IPF), chronic obstructive pulmonary disease (COPD), cystic fibrosis, alpha-1-antitrypsin deficiency, and pulmonary hypertension comprise the majority of those awaiting transplant [2].

 Since the first successful human lung transplant in 1983 [3], there have been significant efforts to improve morbidity and mortality associated with the procedure, particularly as the number of annual lung transplants continues to rise from 837 lung transplants in 1998 to 1,458 in 2007 [4]. One-year survival rates have modestly improved from 73.4% to 80.4% over that time, yet they lag behind those of other solid organ transplant recipients [4].

 Donor lungs in the United States are allocated for use by the lung allocation score (LAS) system, which was instituted in May 2005, in an effort to reduce time and mortality on the transplant waitlist. LAS takes into account a number of factors (Table 1), including diagnosis, functional status, presence of diabetes, assisted ventilation, amount of supplemental oxygen, percent predicted forced vital capacity (FVC), systolic and mean pulmonary artery pressures, mean pulmonary capillary wedge pressure, pCO_2_ levels, six-minute walk distance, and serum creatinine [5, 6]. As recently reviewed by Eberlein et al., the LAS score places twice as much weight on the waitlist urgency measure, which is an estimate of the number of days expected to live on the waitlist, as opposed to the posttransplant survival measure, which estimates the number of days expected to live in the first year after transplant [7, 8]. The LAS system has met its initial goals of decreasing mortality while on the waitlist for transplant, as well as making lung transplant an option for those at greatest need. However, there has been no clear posttransplant benefit in terms of changes in morbidity or mortality, though longer-term data are pending [2, 9]. Further, the usage of the LAS resulted in increased use of resources, morbidity, and mortality in the subgroup of patients with very high LAS [9]. Further insight into other parameters, beyond those in the LAS score, and how they could predict posttransplant outcomes are therefore absolutely necessary to guide future improvements in the field of lung transplantation medicine.

 The role of exercise testing in decisions surrounding appropriateness of listing patients, as well as counseling those undergoing transplant regarding life expectancy, performance status, and posttransplant outcomes, has not yet been fully explored. Exercise testing could provide very useful information that may not be otherwise captured by the LAS.

We performed a National Library of Medicine (Pubmed) search of the English language literature using the following queries “lung transplantation” and “exercise” or “cardiopulmonary stress testing” or “six-minute walk test.” Additional search words were later used for specific topics (e.g., “lung transplantation” and “anemia” or “myopathy”). Manuscripts were included if directly addressed the subject of this review and offered differing points of view or additional explanations.

### 1.2. Six-Minute Walk Test

 The LAS takes into account six minute walk test (6-MWT) performance, one measurement of exercise capacity. The 6-MWT has been used in a number of disease states, including conditions for which patients undergo lung transplantation, such as COPD or IPF, to assess functional status and therapeutic response and define prognosis [18, 19]. One of the first evaluations of the performance of 6-MWT as an assessment in those undergoing lung transplantation was completed in the late 1990s [20]. Results of this retrospective study of 145 patients demonstrated that a pretransplant 6-minute walk distance of less than 400 meters predicted mortality with a sensitivity, specificity, positive predictive value, and negative predictive value of 0.8, 0.27, 0.27, and 0.91, respectively. The authors' conclusion was that the 6-MWT could be useful as a tool to help assess the timing of listing for lung transplantation. In addition, a subsequent retrospective study of 454 patients demonstrated that 6-MWT results—both distance but also presence of desaturation—could be independently associated with mortality for IPF patients awaiting transplant, and, importantly, the test performance was a better predictor of 6-month mortality than spirometry [21]. This demonstrated the relevance of measures of exercise capacity, beyond the data provided by standard pulmonary function tests.

 Despite its incorporation in the LAS, until recently, the 6-MWT has not been evaluated in lung transplant recipients. In their retrospective chart review of 49 patients who had completed 6-MWT six months following transplant, Seoane and colleagues defined normative values for this population and assessed whether test performance predicted mortality [22]. At the 6-month mark, distance walked on the 6-MWT had a normal distribution, with a mean (±SD) of 426 ± 84.5 m (range 240–711 m). At 12 months, for the patients in whom data was available for both 6 months and 12 months, a significant improvement from 348 ± 15 m to 478 ± 14 m was seen (*P* = .0001), with 77% of patients demonstrating an improved walk distance. Despite these improvements, 6-month performance was not a predictor of survival (OR = 1.002).

 To date and to our knowledge, there have not been any studies comparing pre- and post-transplant 6-MWT, so it is not known how pretransplant 6-MWT performance relates to posttransplant functional status and whether there is any significant prognostic information in that relationship. Furthermore, utility of 6-MWT as a marker of functional status, morbidity, or other parameters has not been fully explored. One retrospective study evaluating pretransplant 6-MWT performance of 130 patients concluded that the distance covered was not useful as a marker to predict long-term mortality in the posttransplant setting, as there was no statistically significant difference in survival curves based on distance covered.

 The 6-MWT is the most commonly used test for the assessment of exercise and functional capacity, in part, no doubt, due to the relative ease of administration. Additionally, when compared to other measures of exercise capacity, such as the 2-minute walk test, 12-minute walk test, self-paced walk test, and shuttle walk test, the 6-MWT has been established as well tolerated by patients and more reflective of the activities of daily living [23]. However, performance on a 6-MWT is not a reliable surrogate marker of more traditional indices of exercise capacity—such as cycle ergometry—though some studies have demonstrated utility in some populations, such as COPD patients, who may not be able to complete a CPET [24]. The 6-MWT has several limitation [25]; for instance, it does not determine peak oxygen uptake, and therefore an objective determination of functional capacity and impairment cannot be made, nor can the relative contributing factors that limit exercise be elicited. In addition, the 6-MWT is limited by the so-called “ceiling effect” leaving patients who walked most at baseline with little room for improvement without jogging [26]. Moreover, there is a learning effect when the 6-MWT is administered repeatedly which occurs after the first administration and is maintained for 2 months and occurs to a lesser extent with repeated administration [27]. Though in some clinical situations, the 6-MWT may clearly provide helpful information in terms of functional status. Given these limitations, the American Thoracic Society (ATS) recommendation outlines that 6-MWT data should be complementary to cardiopulmonary exercise testing, and not a substitute [28].

### 1.3. Cardiopulmonary Exercise Test (CPET)

Guidelines regarding routine use of CPET pre- and post-transplant are lacking, which reflects the absence of significant data on how to apply CPET results for optimization of timing of transplant and predicting outcomes. Several studies from the 1990s, described below, have attempted to evaluate the role of CPET in the lung transplant population. Most of these studies (Table 2), however, are limited by small sample sizes, evaluation of a specific respiratory pathology, as well as type of transplant (i.e., single versus bilateral sequential). The lack of generalizability thus makes real-life application of these study results in the clinical setting challenging.

#### 1.3.1. In the Evaluation Phase for Lung Transplantation

The utility of CPET has been evaluated in some specific lung diseases but has not been evaluated on a broader scale in a heterogeneous population. The clinical course of disease progression can be difficult to predict for many chronic respiratory disorders, thereby making selection of the optimal time of transplantation challenging.

**(i) Interstitial Lung Disease (ILD)**

In ILD, there are conflicting reports on the value of different variables on CPET in predicting mortality [10]. In one study of 117 patients with IPF, a peak VO_2_ of less than 8.3 mL/min/kg was associated with an increased risk of subsequent mortality. On adjusted multivariate analysis, VO_2_ max threshold was a more robust predictor of survival than resting PaO_2_ or desaturation below 88% during 6-MWT [10]. However, in this study only 8 patients (6.83%) were below the threshold, but about 46% of patients died. This strongly suggests that while the threshold is informative, it does not help to predict a large part of the mortality in IPF. Surprisingly, not all patients below the threshold peak VO_2_ desaturated below 88% during the 6-MWT, suggesting perhaps that “self pace” allowed patients to trade oxygen saturation for distance walked and perhaps the importance of the composite score distance-saturation product as a more accurate predictor of survival [29].

In a retrospective study of 41 patients, Miki, et al., found that PaO_2_-slope (ΔPaO_2_/ΔVO_2_) was the most useful predictor of survival, independent of age [11]. A PaO_2_-slope equal or less than −60 mmHg/L/min was predictive of decreased survival with a median survival of 1.6 versus 4.5 years. The important aspects of this paper are first that PaO_2_-slope could not be correlated to diffusion capacity of carbon monoxide (DLCO) at rest; second that it suggests potential benefits for pulmonary rehabilitation in IPF (supplemental O_2_, suppressing shallow breathing, etc..). Pulmonary rehabilitation was subsequently shown to improve 6-MWD and fatigue in subjects with IPF [30].

**(ii) Chronic Obstructive Pulmonary Disease (COPD)**

Pulmonary function tests (PFTs) can correlate poorly with exercise performance on CPET for COPD patients, with some patients exceeding exercise limits despite severe impairment on PFTs and others with more surprising limitations than suggested by PFTs. Ortega et al. [12], in their study of 78 stable patients with COPD, showed that the definition of impairment would vary greatly whether an absolute cut-off value for VO_2_ max is used (15 mL/kg/min) or a percent predicted VO_2_ max less than 60%. The absolute number has better specificity (79.5 versus 66.6), and the percent predicted has better sensitivity (64.1% versus 41%) [12]. In a prospective study of 150 male patients with COPD, a multivariate analysis showed that peak VO_2_ expressed as a continuous variable negatively correlated with survival (0.994; 0.992–0.996 *P* < 0.0001). Age was positively correlated with outcome albeit to a lesser degree than VO_2_ max (1.077; 1.010–1.149 *P* = 0.024) [13]. However, this study excluded patients with comorbidities, which are known to play a key role in the outcome of patients with COPD. The same group that examined PaO_2_-slope in IPF [11] conducted a retrospective study of 195 patients and found that PaO_2_-slope correlated to survival more than PFT parameters [14]. A measurement of functional capacity, beyond resting PFTs and 6-MWT, clearly adds valuable information to the care of patients with COPD, allowing improved prognostication and perhaps better timing for transplant listing.

**(iii) Pulmonary Vascular Disease**

In pulmonary hypertension, CPET may be helpful to risk stratify patients into high-risk versus medium- or low-risk [15]. Specifically, in one study of 86 patients with primary pulmonary hypertension, a peak SBP of 120 mm or less and a peak VO_2_ equal to or less than 10.4 mL/kg/min have been established as a powerful predictor of survival threshold. Use of exercise data such as SBP and peak VO_2_ adds additional prognostic information regarding overall survival outcomes, above resting hemodynamics such as right heart catheterization, or imaging such as echocardiogram. Even therapy did not predict survival in this series, strongly suggesting a key role for CPET in the management of patients with pulmonary vascular disease. In a recent review Arena et al. described potential roles for CPET in diagnosed and undiagnosed pulmonary hypertension [31]. Unfortunately, most of the studies included involved small cohorts. Nevertheless, these small studies have consistently observed a relationship between peak VO_2_, VE/VCO_2_ slope, and P_ET_CO_2_ and survival [31].

**(iv) Cystic Fibrosis**

Data suggest that CPET may predict mortality in patients with cystic fibrosis. Specifically, VO_2_ max, VE/VO_2_, and peak work were found to be significant predictors of mortality in a 5-year study of 92 adult patients with cystic fibrosis however FEV1 values appeared to be a better predictor of mortality than peak VO_2_ [16].

In striking contrast, an earlier prospective study of 109 patients with cystic fibrosis found that peak VO_2_ was a significant predictor of mortality, without independent contribution of FEV1. Those with higher levels of aerobic fitness were more than three times likely to survive as compared with those patients with lower levels of fitness, even after adjustment for other risk factors [32]. It remains therefore unclear whether aerobic conditioning or resting lung function is the biggest predictors of outcome in cystic fibrosis. Recent evidence suggesting that muscle function is intrinsically abnormal in cystic fibrosis may perhaps be the link to explain that apparent contradiction [33].

In one series of twenty-eight pediatric patients with cystic fibrosis, rate of decline in peak VO_2_ and peak VO_2_ of less than 32 mL/min/kg was associated with increased mortality; peak VO_2_ of 45 mL/min/kg appeared to be associated with survival [17].

#### 1.3.2. In Posttransplant

In the early 1990s, CPET performance was evaluated in patients who had undergone single and bilateral lung transplantation to evaluate peak VO_2_ [34]. Despite resolution of pretransplant respiratory symptoms and essentially normal pulmonary function in the six bilateral lung transplant recipients—with mild restrictive abnormalities seen in the six patients with single lung transplants—the maximum VO_2_ was markedly reduced one year after transplant. In bilateral lung recipients, VO_2_ maximum was 48.5% of predicted and only 44.2% predicted in single lung recipients. All subjects in this series had significant respiratory reserve, indicating that there was no evidence of ventilatory limitation to exercise in either group.

Results of this study have been confirmed by subsequent studies, one of which further demonstrated that CPET results were unchanged at 2 years after transplant in a cohort of 13 subjects, [35] suggesting that additional improvement does not occur. The peak VO_2_ has been reported at 6 months, with decreases between 9 and 12 months after transplant in a group of eight bilateral transplant recipients as compared to controls [36]. However, other studies suggest that peak VO_2_ may be at 12 or even 24 months [37]. In bilateral lung recipients, peak VO_2_ has been reported as low as 31% of predicted, [38] and as high as 60% [39]. Heart rate and minute ventilation did not appear to be limiting exercise [40]. Interestingly, gains in VO_2_, typically a doubling to about 50% predicted VO_2_, may not be uniform across underlying disease pathologies. One series of 153 subjects evaluated prior to and following transplantation suggested that the most benefit was derived in patients with COPD, emphysema, or alpha-1-antitrypsin, while patients who had interstitial lung disease may have more modest improvements [41]. This may reflect differences in pretransplant level of functioning however, the absolute VO_2_ after transplant again appeared fairly fixed after transplant at 50% predicted VO_2_, regardless of pretransplant capacity. This same series also failed to find a primary pulmonary or cardiac limit to exercise tolerance, with leg fatigue cited as overwhelmingly as reason for termination of exercise. CPET may therefore be useful in identifying causes of poor exercise tolerance in subjects following lung transplant despite improvement of PFT indices.

**(i) Anemia**

Anemia is common subsequent to transplantation of solid organs, with much of the data coming from the renal transplant population. Despite preexisting renal disease in some patients and medication effects on renal function, posttransplantation anemia does not appear to be solely mediated by renal dysfunction [42]. Losses during surgery, medication effects, immune-mediated factors, and different forms of hemolysis can all additionally contribute [43]. Several studies have shown that the percentage drop in VO_2_ max is 2/3 of the percentage drop in hemoglobin [44, 45]. The effect of anemia, even mild anemia, may therefore be profound when considered in conjunction with conditions (discussed below) that might affect tissue oxygen extraction. Understanding the effects of anemia on exercise capacity is important, since transfusion guidelines are increasingly stringent [46], and so other causes of declining functional status in the presence of stable PFTs can be explored.

**(ii) Chronic Muscle Deconditioning and Skeletal Muscle Abnormalities**

Long-term pretransplant debilitation has been felt to play a role in posttransplant exercise performance. However, even in the highest functioning pretransplant group, after transplant, peak VO_2_ reflects pretransplant performance in the 153 patients followed by Bartels prior to and subsequent to transplantation [41]. Underlying disease state does not appear to play a role in absolute peak VO_2_ achieved in another series of fourteen single and eleven bilateral lung transplant recipients [47]. Subgroups of patients, particularly those with COPD, showed greater improvements in VO_2_ max than those patients with IPF [41]. Despite the younger age of patients with cystic fibrosis, age does not appear to play a role in posttransplant peak VO_2_ [41]. This may reflect skeletal muscle abnormalities inherent to CF patients, rather than a posttransplant effect [48].

Suggesting that there may be other factors at play in other populations, Miyoshi's study following six single and six bilateral lung transplant recipients suggested that peak VO_2_ is not significantly different after lung transplant as compared to heart-lung recipients [34]. Indeed, consistent findings clearly show that in posttransplant patients, peak VO_2_ is not limited by ventilatory reserve, but instead, systemic oxygen extraction is abnormal [49, 50]. Specifically, peak cardiac output is normal, as much as 89% of predicted; there is even evidence of beneficial right heart remodeling seen after bilateral lung transplantation based on a prospective study following twenty subjects before and after transplantation [37]. Despite this, however, arterial-mixed venous oxygen content difference at peak exercise was only half of expected, primarily due to inability of venous oxygen saturation to decline normally.

In one series comparing exercise capacity of nine lung transplant recipients with that of eight healthy volunteers, ventilation, oxygen saturation, and mild anemia did not appear to account for decreased peak VO_2_ as compared to healthy volunteers. Instead, quadriceps muscle pH and phosphorylation potential appeared to be reduced following transplant, suggesting that abnormalities of skeletal muscle oxidative capacity may be playing a role in decreased peak VO_2_ after transplant [51]. Indeed, another study of twenty-five subjects confirmed a correlation between quadriceps muscle force after transplant and exercise capacity [52]. The importance of posttransplant exercise and pulmonary rehabilitation cannot be overstated, with multiple studies showing positive effects of rehabilitation on skeletal muscle function and respiratory status, immediately after transplant and in long-term survivors of lung transplant, without clear benefit for inpatient or outpatient rehabilitation [53–55].

**(iii) Medications**

Proximal myopathy due to corticosteroids and peripheral skeletal muscle abnormalities due to cyclosporine have all been proposed likely contributing factors to reduced exercise capacity [56]. In particular, even a short course of 5 days of corticosteroids, as used for acute rejection, may have profound effects on respiratory and skeletal muscle, with up to 45% of patients developing acute generalized weakness as measured in one series of thirteen subjects after lung transplantation [57]. Another study evaluating muscle size and strength in six COPD patients compared to six patients who had undergone single-lung transplantation for COPD found that despite similar strength and muscle size, muscle endurance was lower in the transplant population, pointing towards a possible effect of immunosuppressant medications, as suggested by animal data [58, 59]. The calcineurin signaling pathway may play a role in signaling transition from fast-to-slow skeletal muscle fiber-type transition [60]. Inhibition of this pathway, therefore, could have important implications in terms of peak work and exercise endurance in the posttransplant setting. This is of important clinical implications, particularly in patients whose lung function improves after transplant but their exercise tolerance does not correlate with the improvement in lung function.

**(iv) Effects of Native Lung in COPD**

CPET has also been evaluated in patients who have undergone single lung transplantation for COPD [61]. VO_2_ max in six single lung recipients with underlying COPD was similar to that of six single lung recipients with underlying IPF, without evidence of ventilatory limitations to exercise. These findings alleviate concerns over the hyperinflated native lung causing decreased tidal volume, leading to ventilation perfusion mismatch and impaired gas exchange. This is of critical importance, since it should alleviate concerns over single lung transplant in subjects with COPD [62] and should lead clinicians to focus on other causes of lack of symptomatic improvement of subjects with COPD following lung transplant.

## 2. Summary

 There clearly is a need for further prospective systematic investigation of the role of CPET in the pre- and post- lung transplant period. The above findings suggest that CPET may provide useful information prior to transplantation in various disease states, including risk stratification and prediction of mortality, in some cases beyond that of pulmonary function tests and the 6-MWT. Additionally, usage of CPET after transplant has repeatedly demonstrated that despite improvement in lung function, the primary limitation to exercise appears to be at the level of oxygen extraction. If these contributing factors are better elucidated, further improvements in outcomes may occur, either by further optimizing factors in pretransplant setting or better stratifying risk after transplant.

## Figures and Tables

**Table 1:** Components of the Lung Allocation Score.

Date of birth
Height
Weight
Lung diagnosis
Functional status
Performs activities of daily living
Some assistance
Total assistance
No assistance
Diabetes
Dependency unknown
Not diabetic
Insulin dependent
Not insulin dependent
Mechanical ventilation
BiPAP
CPAP
Continuous mechanical
Intermittent mechanical
No assisted ventilation
Supplemental O_2_ requirements
At rest
At night
With exercise
Not needed
Amount (liters)FiO_2_(%)
Forced vital capacity (%predicted)
Pulmonary artery systolic pressure (mmHg)
Mean pulmonary artery pressure
Pulmonary capillary wedge pressure
Current pCO_2_
Highest pCO_2_
Lowest pCO_2_
Change in pCO_2_ (%)
Six minute walk distance (feet)
Serum creatinine (mg/dL)

**Table 2:** CPET indicators that correlate with outcome.

Disease	CPET outcome variable	Reference
Idiopathic pulmonary fibrosis	VO_2_ ≤ 8.3 mL/min/kg	[10]
	PaO_2_ slope ≤ −60 mmHg/L/min	[11]
Chronic obstructive pulmonary disease	VO_2_ max (<15 mL/min/kg)	[12]
	VO_2_ max < 60% predicted	[12]
	VO_2_ max	[13]
	PaO_2_ slope ≤ −80 mmHg/L/min	[14]
Pulmonary vascular disease	peak SBP ≤ 120 mm or less and	[15]
	peak VO_2_ ≤ 10.4 mL/kg/min	
Cystic fibrosis	VO_2_ max,VE/VO_2_, peak work	[16]
	VO_2_ of 45 mL/min/kg	[17]

## References

[1] http://optn.transplant.hrsa.gov/data/default.asp.

[2] Yusen RD, Shearon TH, Qian Y (2010). Lung transplantation in the United States, 1999–2008: special feature. *American Journal of Transplantation*.

[3] Cooper JD, Ginsberg RJ, Goldberg M (1986). Unilateral lung transplantation for pulmonary fibrosis. *The New England Journal of Medicine*.

[4] Department of Health and Human Services HRaSA, Healthcare Systems Bureau, Division of Transportation, Rockville, Md, USA (2009). *Annual Report of the U.S. Organ Procurement and Transplantation Network and teh Scientific Registry of Transplant Recipients: Transplant Data 1999–2008*.

[5] Egan TM, Murray S, Bustami RT (2006). Development of the new lung allocation system in the United States. *American Journal of Transplantation*.

[6] Lung Allocation Score Calculator http://optn.transplant.hrsa.gov/resources/professionalResources.asp.

[7] Eberlein M, Garrity ER, Orens JB (2011). Lung allocation in the United States. *Clinics in Chest Medicine*.

[8] A Guide to Calculating the Lung Allocation Score http://www.unos.org/docs/lung_allocation_score.pdf.

[9] Merlo CA, Weiss ES, Orens JB (2009). Impact of U.S. Lung Allocation Score on Survival After Lung Transplantation. *Journal of Heart and Lung Transplantation*.

[10] Fell CD, Liu LX, Motika C (2009). The prognostic value of cardiopulmonary exercise testing in idiopathic pulmonary fibrosis. *American Journal of Respiratory and Critical Care Medicine*.

[11] Miki K, Maekura R, Hiraga T (2003). Impairments and prognostic factors for survival in patients with idiopathic pulmonary fibrosis. *Respiratory Medicine*.

[12] Ortega F, Montemayor T, Sanchez A, Cabello F, Castillo J (1994). Role of cardiopulmonary exercise testing and the criteria used to determine disability in patients with severe chronic obstructive pulmonary disease. *American Journal of Respiratory and Critical Care Medicine*.

[13] Oga T, Nishimura K, Tsukino M, Sato S, Hajiro T (2003). Analysis of the factors related to mortality in chronic obstructive pulmonary disease: role of exercise capacity and health status.. *American Journal of Respiratory and Critical Care Medicine*.

[14] Hiraga T, Maekura R, Okuda Y (2003). Prognostic predictors for survival in patients with COPD using cardiopulmonary exercise testing. *Clinical Physiology and Functional Imaging*.

[15] Wensel R, Opitz CF, Anker SD (2002). Assessment of survival in patients with primary pulmonary hypertension: importance of cardiopulmonary exercise testing. *Circulation*.

[16] Moorcroft AJ, Dodd ME, Webb AK (1997). Exercise testing and prognosis in adult cystic fibrosis. *Thorax*.

[17] Pianosi P, LeBlanc J, Almudevar A (2005). Peak oxygen uptake and mortality in children with cystic fibrosis. *Thorax*.

[18] Flaherty KR, Andrei AC, Murray S (2006). Idiopathic pulmonary fibrosis: prognostic value of changes in physiology and six-minute-walk test. *American Journal of Respiratory and Critical Care Medicine*.

[19] Sciurba F, Criner GJ, Lee SM (2003). Six-minute walk distance in chronic obstructive pulmonary disease: reproducibility and effect of walking course layout and length. *American Journal of Respiratory and Critical Care Medicine*.

[20] Kadikar A, Maurer J, Kesten S (1997). The six-minute walk test: a guide to assessment for lung transplantation. *Journal of Heart and Lung Transplantation*.

[21] Lederer DJ, Arcasoy SM, Wilt JS, D’Ovidio F, Sonett JR, Kawut SM (2006). Six-minute-walk distance predicts waiting list survival in idiopathic pulmonary fibrosis. *American Journal of Respiratory and Critical Care Medicine*.

[22] Seoane L, Alex S, Pirtle C (2010). Utility of the 6-minute walk test following lung transplantation. *Ochsner Journal*.

[23] Solway S, Brooks D, Lacasse Y, Thomas S (2001). A qualitative systematic overview of the measurement properties of functional walk tests used in the cardiorespiratory domain. *Chest*.

[24] Cote CG, Pinto-Plata V, Kasprzyk K, Dordelly LJ, Celli BR (2007). The 6-min walk distance, peak oxygen uptake, and mortality in COPD. *Chest*.

[25] Heresi GA, Dweik RA (2011). Strengths and limitations of the six-minute-walk test: a model biomarker study in idiopathic pulmonary fibrosis. *American Journal of Respiratory and Critical Care Medicine*.

[26] Frost AE, Langleben D, Oudiz R (2005). The 6-min walk test (6MW) as an efficacy endpoint in pulmonary arterial hypertension clinical trials: demonstration of a ceiling effect. *Vascular Pharmacology*.

[27] Wu G, Sanderson B, Bittner V (2003). The 6-minute walk test: how important is the learning effect?. *American Heart Journal*.

[28] Brooksa D, Solwayb S (2002). ATS statement: guidelines for the six-minute walk test. *American Journal of Respiratory and Critical Care Medicine*.

[29] Lettieri CJ, Nathan SD, Browning RF, Barnett SD, Ahmad S, Shorr AF (2006). The distance-saturation product predicts mortality in idiopathic pulmonary fibrosis. *Respiratory Medicine*.

[30] Swigris JJ, Fairclough DL, Morrison M (2011). Benefits of pulmonary rehabilitation in idiopathic pulmonary fibrosis. *Respiratory Care*.

[31] Arena R, Lavie CJ, Milani RV, Myers J, Guazzi M (2010). Cardiopulmonary exercise testing in patients with pulmonary arterial hypertension: an evidence-based review. *Journal of Heart and Lung Transplantation*.

[32] Nixon PA, Orenstein DM, Kelsey SF, Doershuk CF (1992). The prognostic value of exercise testing in patients with cystic fibrosis. *The New England Journal of Medicine*.

[33] Selvadurai HC, Allen J, Sachinwalla T, Macauley J, Blimkie CJ, Van Asperen PP (2003). Muscle function and resting energy expenditure in female athletes with cystic fibrosis. *American Journal of Respiratory and Critical Care Medicine*.

[34] Miyoshi S, Trulock EP, Schaefers HJ, Hsieh CM, Patterson GA, Cooper JD (1990). Cardiopulmonary exercise testing after single and double lung transplantation. *Chest*.

[35] Williams TJ, Patterson GA, McClean PA, Zamel N, Maurer JR (1992). Maximal exercise testing in single and double lung transplant recipients. *American Review of Respiratory Disease*.

[36] Pellegrino R, Rodarte JR, Frost AE, Reid MB (1998). Breathing by double-lung recipients during exercise: response to expiratory threshold loading. *American Journal of Respiratory and Critical Care Medicine*.

[37] Habedank D, Ewert R, Hummel M (2011). The effects of bilateral lung transplantation on ventilatory efficiency, oxygen uptake and the right heart: a two-yr follow-up. *Clinical Transplantation*.

[38] Oelberg DA, Systrom DM, Markowitz DH (1998). Exercise performance in cystic fibrosis before and after bilateral lung transplantation. *Journal of Heart and Lung Transplantation*.

[39] Levy RD, Ernst P, Levine SM (1993). Exercise performance after lung transplantation. *Journal of Heart and Lung Transplantation*.

[40] Schwaiblmair M, Reichenspurner H, Müller C (1999). Cardiopulmonary exercise testing before and after lung and heart-lung transplantation. *American Journal of Respiratory and Critical Care Medicine*.

[41] Bartels MN, Armstrong HF, Gerardo RE (2011). Evaluation of pulmonary function and exercise performance by cardiopulmonary exercise testing before and after lung transplantation. *Chest*.

[42] Blosser CD, Bloom RD (2010). Posttransplant anemia in solid organ recipients. *Transplantation Reviews*.

[43] Modrykamien A (2010). Anemia post-lung transplantation: mechanisms and approach to diagnosis. *Chronic Respiratory Disease*.

[44] Woodson RD (1984). Hemoglobin concentration and exercise capacity. *American Review of Respiratory Disease*.

[45] Woodson RD, Wills RE, Lenfant C (1978). Effect of acute and established anemia on O2 transport at rest, submaximal and maximal work. *Journal of Applied Physiology Respiratory Environmental and Exercise Physiology*.

[46] Murphy MF, Wallington TB, Kelsey P (2001). Guidelines for the clinical use of red cell transfusions. *British Journal of Haematology*.

[47] Orens JB, Becker FS, Lynch JP, Christensen PJ, Deeb GM, Martinez FJ (1995). Cardiopulmonary exercise testing following allogeneic lung transplantation for different underlying disease states. *Chest*.

[48] Vallier JM, Gruet M, Mely L, Pensini M, Brisswalter J (2011). Neuromuscular fatigue after maximal exercise in patients with cystic fibrosis. *Journal of Electromyography and Kinesiology*.

[49] Systrom DM, Pappagianopoulos P, Fishman RS, Wain JC, Ginns LC (1998). Determinants of abnormal maximum oxygen uptake after lung transplantation for chronic obstructive pulmonary disease. *Journal of Heart and Lung Transplantation*.

[50] Tirdel GB, Girgis R, Fishman RS, Theodore J (1998). Metabolic myopathy as a cause of the exercise limitation in lung transplant recipients. *Journal of Heart and Lung Transplantation*.

[51] Evans AB, Al-Himyary AJ, Hrovat MI (1997). Abnormal skeletal muscle oxidative capacity after lung transplantation by 31P-MRS. *American Journal of Respiratory and Critical Care Medicine*.

[52] Reinsma GD, ten Hacken NHT, Grevink RG, van der Bij W, Koëter GH, van Weert E (2006). Limiting factors of exercise performance 1 year after lung transplantation. *Journal of Heart and Lung Transplantation*.

[53] Ihle F, Neurohr C, Huppmann P (2011). Effect of inpatient rehabilitation on quality of life and exercise capacity in long-term lung transplant survivors: a prospective, randomized study. *Journal of Heart and Lung Transplantation*.

[54] Maury G, Langer D, Verleden G (2008). Skeletal muscle force and functional exercise tolerance before and after lung transplantation: a cohort study. *American Journal of Transplantation*.

[55] Munro PE, Holland AE, Bailey M, Button BM, Snell GI (2009). Pulmonary Rehabilitation Following Lung Transplantation. *Transplantation Proceedings*.

[56] Kawut SM, O’Shea MK, Bartels MN, Wilt JS, Sonett JR, Arcasoy SM (2005). Exercise testing determines survival in patients with diffuse parenchymal lung disease evaluated for lung transplantation. *Respiratory Medicine*.

[57] Nava S, Fracchia C, Callegari G, Ambrosino N, Barbarito N, Felicetti G (2002). Weakness of respiratory and skeletal muscles after a short course of steroids in patients with acute lung rejection. *European Respiratory Journal*.

[58] Mathur S, Levy RD, Reid WD (2008). Skeletal muscle strength and endurance in recipients of lung transplants. *Cardiopulmonary Physical Therapy Journal*.

[59] Pandorf CE, Jiang WH, Qin AX, Bodell PW, Baldwin KM, Haddad F (2009). Calcineurin plays a modulatory role in loading-induced regulation of type I myosin heavy chain gene expression in slow skeletal muscle. *American Journal of Physiology*.

[60] Miyazaki M, Hitomi Y, Kizaki T, Ohno H, Haga S, Takemasa T (2004). Contribution of the calcineurin signaling pathway to overload-induced skeletal muscle fiber-type transition. *Journal of Physiology and Pharmacology*.

[61] Gibbons WJ, Levine SM, Bryan CL (1991). Cardiopulmonary exercise responses after single lung transplantation for severe obstructive lung disease. *Chest*.

[62] Munson JC, Christie JD, Halpern SD (2011). The societal impact of single versus bilateral lung transplantation for chronic obstructive pulmonary disease. *American Journal of Respiratory and Critical Care Medicine*.

